# Reliability Analysis Method of Rotating Machinery Based on Conditional Random Field

**DOI:** 10.1155/2022/7326730

**Published:** 2022-10-03

**Authors:** Hongmei Zheng, Xiaoli Qiao

**Affiliations:** Department of Information and Electromechanical Engineering, Shaoxing University Yuanpei College, Shaoxing 312000, China

## Abstract

Rotating machinery is indispensable mechanical equipment in modern industrial production. However, rotating machinery is usually under heavy load. Due to the complexity of its structure and the severity of its working conditions, it is urgent to find effective condition monitoring methods and fault maintenance strategies for its safe and reliable operation. The conditional random field is derived from the maximum entropy model, which solves the problem of label bias and improves the convergence speed of model training. Combining Kriging theory and random field theory, this study proposes a three-dimensional conditional random field generation method based on failure time, applies this method to the comparison of measured data and other nonconditional random fields, and then analyzes the failure probability of rotating machinery in the failure process by combining the numerical calculation results and reliability theory. It is found that the conditional random field generation method can effectively describe the spatial variability of rotating machinery parameters. Compared with the nonconditional random field, the reliability index of rotating machinery failure time is improved by 0.8823, so the conditional random field can better describe the reliability of rotating machinery.

## 1. Introduction

Rotating machinery is mainly composed of rotors, stators, bearings, couplings, housings, etc. [1]. The working state drives the movement of other components to realize the predetermined mechanical movement, which is a kind of modern industrial production. Rotating machinery is an indispensable main equipment in industrial machinery because of its unique properties and functions [2]. Typical rotating machinery includes gearboxes, reducers, steam turbines, gas turbines, fans, generators, and engines, which are widely used in electric power, petrochemical, metallurgy, automobile manufacturing, aerospace, and other sectors [3].

Rotating machinery is usually in the continuous operation state of heavy load and high speed, and the failure of different failure forms occurred in rotating machinery; it is easy to affect its normal work [4]. Once these core components fail, the light ones will affect the use, and the serious ones will cause downtime or even casualties. As key equipment widely used in all walks of life, rotating machinery plays a pivotal role in production [5, 6]. At the same time, due to the complexity of its structure and the harsh working conditions, it is urgently needed for its safety. Reliable operation finds effective condition monitoring methods and fault maintenance strategies. Therefore, failure analysis and reliability analysis of rotating machinery have become a very critical part of system design and maintenance.

Fault diagnosis of mechanical equipment is a scientific technology for monitoring, diagnosing, and predicting the status of continuous running equipment and ensuring the safe operation of mechanical equipment. Its prominent feature is the close combination of theoretical research and engineering practice. It is an advanced technology for recording and analyzing the equipment status and identifying and alarming the abnormal status by using various measurements and monitoring methods. The application of this technology can find the fault state of mechanical equipment in time, avoid the occurrence of catastrophic events, and avoid the economic loss caused by insufficient or excessive maintenance, which has great economic benefits. As the most important component in the mechanical system, such as induction motor, it is the main device to drive various mechanical equipment. It is widely used in various mechanical equipment, and its operation reliability and safety should be higher. Academician Qu Liangsheng has summarized that mechanical equipment is divided into three basic parts: gear, bearing, and rotating shaft system, and rotating parts such as bearing and gear are also widely used in various mechanical systems. According to the research, it is found that the bearing damage fault accounts for about 40% of the faults of rotating machinery, and the fault caused by gear failure accounts for about 10.3%. Therefore, it is of great significance to find out the fault conditions in the rotating machinery in time and conduct accurate fault diagnosis and maintenance for the rotating machinery to ensure the safe and stable operation of the production system and reduce the probability of catastrophic accidents [3].

The fault diagnosis technology of rotating machinery is an interdisciplinary subject combining with practice and integrating computer technology, mathematical theory, electronic technology, signal processing technology, and other modern technologies. Its essence can be regarded as the problem of pattern recognition and classification, including signal detection, feature extraction, state recognition, and decision diagnosis. Although some achievements have been made in the fault diagnosis of rotating machinery in recent years, for the monitoring and diagnosis of modern complex mechanical equipment, a large number of nonlinear high-dimensional data information will be generated by the detection of multiple sensors, while the traditional fault diagnosis method that relies on manual feature extraction will generate a huge workload when analyzing and processing these data, reducing the efficiency of fault diagnosis, The accuracy of fault diagnosis results depends on the effectiveness of feature extraction, and effective feature extraction technology needs the prior knowledge of the research object as the basis. Therefore, feature extraction technology is a key issue in the development of traditional mechanical fault diagnosis technology. In view of the current complex big data research background, the research on intelligent mechanical fault diagnosis methods can reduce the degree of manual intervention, improve the efficiency of fault diagnosis, and help to ensure the safe and reliable operation of rotating machinery.

Intelligent manufacturing engineering has become an important development trend of the manufacturing industry. Intelligent manufacturing is based on the deep integration of the new generation of information and communication technology and advanced manufacturing technology. It runs through all links of manufacturing activities such as design, production, management, and service. It has the characteristics of self-perception, self-learning, self-determination, self-implementation, and self-adaptation. The development of Intelligent Manufacturing in China has made certain achievements, and the key technology and equipment represented by high-end CNC machine tools, industrial robots, and intelligent instruments have made positive progress. The intellectualization of industrial manufacturing also puts forward intelligent requirements for the fault diagnosis system of rotating machinery. The intelligent fault diagnosis is realized by means of big data-driven knowledge learning and self-help intelligent system, which helps to improve the intelligent level of major equipment. The new generation of artificial intelligence technology based on deep learning is an effective method to realize intelligent fault diagnosis through deep feature mining of data and independent knowledge learning. According to the characteristics of large amount and high dimension of mechanical equipment monitoring data, it is the key development direction in the future to realize targeted intelligent diagnosis model design and explore intelligent diagnosis methods based on multisource data fusion and deep feature extraction.

With the development of computer science, intelligent methods such as artificial intelligence, pattern recognition, and machine learning have been continuously applied to mechanical fault diagnosis tasks [7], such as neural network (NN), support vector machine (Support Vector Machine, SVM), Hidden Markov Model (HMM), genetic algorithm, fuzzy theory, and manifold learning, and other intelligent pattern recognition technologies have achieved good results. Pan et al. proposed a multiclass fuzzy support matrix classifier for rolling bearing fault diagnosis [8]. Cerrada et al. established a model for feature selection and model tuning applied to fault severity diagnosis in Spur Gearboxes [9]. Xu et al. proposed a K-nearest neighbor-based method [10]. Chen et al. proposed a supervised Fuzzy C Means Clustering (SFCM) to diagnose rotating machinery [11]. Shen et al. of Georgia Institute of Technology used fuzzy neural network and Bayesian algorithm to predict the health status of mechanical systems [12]. Sun et al. used wavelet transform to extract feature information, combined with distance evaluation technology to reduce the dimension of feature space, and finally used the support vector regression method to construct rotating machinery fault identification method [13]. Lafferty and Mccallum proposed a method of augmented popular learning using kernel sparse representation to construct neighborhood and successfully applied to gearbox health monitoring [14]. The artificial intelligence method enables the computer to have the ability of feature learning, replaces the process of manual feature extraction, and combines feature learning and fault identification and classification into an organic whole, thereby realizing intelligent fault diagnosis and reducing the impact of manual participation on the fault diagnosis system. With the continuous attempts in the field of machine learning in recent years, deep learning technology has developed rapidly, and intelligent mechanical fault diagnosis technology based on deep learning has also gradually developed.

The conditional random field model is a machine learning model based on the hidden Markov model. It simulates conditional probability and is mainly used to label and segment serialized data. It was first used for sequence labeling. At present, it has been widely used in various fields including word processing, biomedicine, computer vision, social intelligence, intrusion detection, information extraction, speech processing, and other fields. In this study, the method of establishing conditional random field model is used to analyze the reliability of rotating machinery.

## 2. Existing Problems of Fault Analysis for Rotating Machinery

### 2.1. Intelligent Fault Diagnosis Method Based on Nonartificial Feature Extraction

Traditional fault diagnosis methods of rotating machinery often include feature extraction process based on manual operation., Personnel with certain prior knowledge are required to preprocess the sensor signals of the operating equipment, from the original to extract and select characteristic information that can effectively reflect the operating state of the equipment from the sensor signals. The process of fault identification depends on the operator's understanding of a sensor signal. Background knowledge and the effectiveness of feature extraction and selection methods will greatly affect the quality of feature extraction and selection. Influence the accuracy of final fault diagnosis, and for different rotating mechanical components in different environments, even in suitable feature extraction, feature selection methods are different between different types of sensor information. In the line monitoring and fault diagnosis tasks, the requirements for operators are higher. From methods based on artificial feature extraction, in the past, it will add some uncertainty to the fault diagnosis system, and the success or failure of mechanical equipment fault diagnosis depends on the characteristics. Good or bad, it is, therefore, desirable to obtain an intelligent mechanical fault diagnosis method that does not rely on artificial feature extraction. Using machine learning and deep learning, a fault diagnosis model based on feature learning is constructed, and the features are extracted. Combined with fault identification, it becomes a complete fault diagnosis system. The system can learn from the data samples and automatically adjust the network model parameters according to the purpose of accurate fault identification so that the model can change from the original sensing.

### 2.2. Deep Network Fault Diagnosis Method Based on Multisensor Information Fusion

In the current research situation, most of the mechanical fault diagnosis methods are based on a single sensor signal. For example, for motor fault diagnosis, current signal is often used for feature extraction and recognition analysis and identification. For fault diagnosis of bearing gearbox, data analysis and processing are generally based on vibration signals. For a simple mechanical system, a single sensor information can meet the requirements of fault diagnosis and identification. However, for complex mechanical equipment systems, the mechanical equipment characteristics are contained in a single type of sensor information. The information is limited, and the presence of noise signal, harmonic signal, and other influencing factors will greatly affect the fault diagnosis the recognition effect of the system; at this time, we need to integrate various and multidirectional sensor information to make different sensors; the information can complement each other and expand the feature space, so as to improve the robustness of the whole fault diagnosis model to achieve stable and accurate fault diagnosis of rotating machinery.

### 2.3. Fault Diagnosis Method Based on Small Sample Training

The design of fault diagnosis system based on deep learning technology often depends on the quality of training data set and needs class; the balanced large-scale labeled data can obtain a more reasonable network model only after sufficient training and learning of the network model data modeling to achieve the final fault diagnosis. However, in the actual application process, for the rotating machinery in terms of line status, most of the mechanical equipment operates under normal status, and sufficient normal status data samples can be obtained. However, the fault status of mechanical equipment is often a small probability event; corresponding to the collected fault status, the samples are also limited, and it is a time-consuming work to label the fault status. About deep network model, training needs to rely on class balanced datasets. For class unbalanced datasets, size of data often depends on the minimum amount of data, which also limits the deep learning technology in the field of fault diagnosis. Therefore, we hope to make use of the generation model in the field of deep learning; and the data increase strong technology to expand the training dataset, supplement such unbalanced data samples, and increase the data space of the training set; the reasonable fault diagnosis model is obtained by training the layer network model, and the learning modeling of small sample dataset is realized.

### 2.4. Design of Fault Diagnosis Model Based on Large Deep Network Structure

At present, intelligent mechanical fault diagnosis models based on deep learning often contain only a few hidden layers; the deep neural network structure with no more than five hidden layers is applied to the fault diagnosis task of rotating machinery. On the one hand, it is difficult to collect labeled fault samples due to the limited data sets in the field of mechanical fault diagnosis, especially the small sample events in some fault states. When the deep network model has more hidden layers, it is very difficult to train large-scale networks based on small sample data. On the other hand, large-scale deep network models often contain more model parameters and super parameter combinations. Training large-scale deep network models to complete convergence requires not only a large amount of labeled data but also a large amount of training time, which increases the cost of fault diagnosis models. However, for the network model with few hidden layers, its feature learning ability is limited, and it cannot effectively model the operation state of mechanical equipment under complex working conditions. The large-scale deep network model with multilayer hidden layers has higher data processing ability and nonlinearity mapping ability, better model generalization ability, and stable model expressiveness. The feature information extracted from the high level is also more abstract and conducive to the distinction between fault states. Therefore, it is hoped to propose a general-purpose mechanical fault diagnosis model based on large-scale deep network model to achieve rapid and accurate fault identification.

### 2.5. Application of Iterative Learning to Update Weight in Signal

There are many deep learning models, and there seems to be no clear way to solve a problem in the field of fault diagnosis. Most of the existing studies are modified according to the existing deep learning model. Whether the idea of deep learning can be applied and a more suitable deep learning model can be designed according to the characteristics of vibration signals may be a direction of future research. When traditional methods solve the problem of fault diagnosis, they are often the combination of multiple methods: first is extracting features and then is pattern recognition. Deep learning brings these processes together and completes “end-to-end” fault diagnosis. Is this the most perfect solution? Should feature extraction methods be eliminated?

## 3. Establishment of Random Field for Rotating Machinery Condition

### 3.1. Basic Theory of Conditional Random Fields

In 2001, Lafferty et al. first proposed the conditional random field (CRF) theory and gave its definition, pointing out that it not only does not require the independence assumption of generative models (such as HMM) but also overcomes the maximum entropy Markov model (MEMM) label bias problem [15].

The basic definition of conditional random field: if *G*=(*V*, *E*) represents an undirected graph, *Y*=(*Y*_*v*_)_*vϵV*_, the elements in *Y* correspond to the vertices in the undirected graph *G* one-to-one, and *X* is the observed value of *Y*. When *X* is known, if the conditional probability distribution of the random variable obeys the Markov property, *P*=(*Y*_*v*_|*X*, *Y*_*w*_, *w* ≠ *v*)=*P*=(*Y*_*v*_|*X*, *Y*_*w*_, *w* ~ *v*), and *w* ~ *v* represents (*w*, *v*), the neighbor node of the undirected graph *G*, and (*X*, *Y*) is said to be a conditional random field.

**Figure 1:**
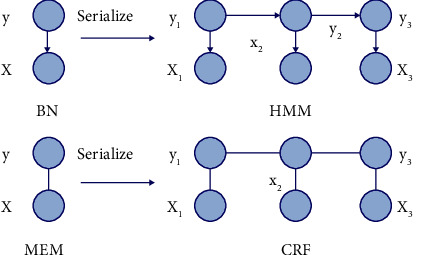
Probability diagram representation of a similar model.

A CRF is essentially a Markov Random Field (MRF) given a set of observations. MRF is a random field restricted by the Markov property. The Markov property means that, for any random variable in (MRF), the distribution of the variable under all other variables in the given field is equivalent to that in the given field. The distribution of the variable under the neighbor node of the variable: the main idea is that the variable far away from the variable has little influence on the current variable. The correlation between conditional random field and other fields is shown in Figure 1.

The Hammersley–Clifford [16] theorem proves that the Gibbs distribution and the MRF are equivalent. The core idea of Gibbs distribution is that the probability of an undirected graph can be factored, that is to say, the joint probability of the entire graph can be expressed as the product of the potential functions on all the largest groups contained in the graph, where *V* represents the set of all nodes in the graph and *E* represents the set of all adjacent edges in the graph. In order to ensure that the product of potential functions satisfies the probability axiom, a normalization factor *Z* is introduced to obtain the joint probability formula of MRF as follows:

(1) $$\begin{array}{c} P\left(x\right)=\frac{1}{Z}\underset{c\epsilon C}{\prod }\Psi_{C}\left(X_{C}\right) \end{array}$$

where *C* represents the set of the largest clique *c*.

If there are corresponding observations for each random variable in a given, then the MRF distribution when these observations are known is the conditional distribution, and the MRF can also be called CRF. The graph corresponding to CRF is an undirected graph model, so CRF is essentially an MRF given a set of observations, so there are

(2) $$\begin{array}{c} P\left(y|x\right)=\frac{P\left(x,y\right)}{P\left(x\right)}=\frac{P\left(x,y\right)}{\underset{y^{`}}{\sum }P\left(y^{`},x\right)}=\frac{1/Z\prod_{c\epsilon C}\Psi_{C}\left(x,y_{c}\right)}{1/Z\underset{y^{`}}{\sum }1/Z\underset{c\epsilon C}{\prod }\Psi_{C}\left(x,y_{c}^{`}\right)}=\frac{\prod_{c\epsilon C}\Psi_{C}\left(x,y_{c}\right)}{\underset{y^{`}}{\sum }1/Z\underset{c\epsilon C}{\prod }\Psi_{C}\left(x,y_{c}^{`}\right)} \end{array}$$

where *Z* is a normalization constant, Ψ_*C*_(*x*, *y*_*c*_) is the potential function of the *c*th clique, and *y*_*c*_ represents the random variable of the *c*th clique.

Therefore, the general form of a conditional random field is

(3) $$\begin{array}{c} \begin{array}{c} P\left(y|x\right)=\frac{1}{Z\left(x\right)}\prod_{c\epsilon C}\Psi_{C}\left(x,y_{c}\right)\\ Z\left(x\right)=\underset{y^{`}}{\sum }\prod_{c\epsilon C}\Psi_{C}\left(x,y_{c}^{`}\right) \end{array}\text{.} \end{array}$$

Lafferty's choice of the CRF potential function is largely influenced by the maximum entropy model, and each potential function is defined as follows:

(4) $$\begin{array}{c} \Psi_{C}\left(x,y_{c}\right)=\exp\;\left(\underset{k}{\sum }\theta_{k}f_{k}\left(c,y_{c},x\right)\right) \end{array}$$

where *f*_*k*_ is a Boolean feature function and *θ*_*k*_ is the weight corresponding to the feature function.

Therefore, *P*(*y|x*) can be expressed as

(5) $$\begin{array}{c} \begin{array}{c} P\left(y|x\right)=\exp\;\left(\underset{k}{\sum }\theta_{k}f_{k}\left(c,y_{c},x\right)\right)\\ Z\left(x\right)=\underset{y^{`}}{\sum }\left(\;\exp\underset{c\epsilon C}{\sum }\left.\underset{k}{\sum }\theta_{k}f_{k}\left(c\right.,y_{c},\left.x\right)\right)\right. \end{array}\text{.} \end{array}$$

### 3.2. Kriging Theory

In order to make the prediction model for rotating machinery established by the conditional random field more accurate, the Kriging theory was introduced to improve the basic conditional random field [17]. The Kriging method is a frequently used optimal linear unbiased estimation method, which can obtain the predicted value by weighting the existing data linearly. Since the error of this method is as close to 0 as possible, the Kriging method is not biased. The most important thing is that compared with other forecasting methods, the Kriging method has the smallest variance of forecast error and is the best linear unbiased estimation method [18].

In the Kriging method, all samples will participate in the estimation of unknown points, and in the case that the variation function increases with distance, the samples close to the unknown point have more influence. In practical application, in order to highlight the local characteristics of the random field, the influence range of the point can be set artificially, without considering the influence of samples outside the range. This property also means that if there is no adjacent sample somewhere in the random field, the Kriging variance will increase, so the Kriging method is only applicable to the interpolation of lattice data. The extrapolation results of ordinary Kriging approach the sample average value with the increase of the point distance. Pan Kriging will extrapolate the drift infinitely during extrapolation.

The estimated *x*_0_ value at the location *Z*^*∗*^(*x*_0_) can be expressed as

(6) $$\begin{array}{c} Z^{*}\left(x_{0}\right)=\overset{n}{\underset{i=1}{\sum }}\lambda_{i}Z\left(x_{i}\right) \end{array}$$

where *Z*(*x*_*i*_) is the measurement value at the position *x*_*i*_ and *λ*_*i*_ is the residual weight of the measurement value; then, the average value of the estimated error is

(7) $$\begin{array}{c} E\left[Z^{*}\left(x_{0}\right)-Z\left(x_{0}\right)\right]=E\left[\overset{n}{\underset{i=1}{\sum }}\lambda_{i}Z\left(x_{i}\right)-Z\left(x_{0}\right)\right]=E\left[Z\left(x_{0}\right)\right]\left(\overset{n}{\underset{i=1}{\sum }}\lambda_{i}-1\right)\text{.} \end{array}$$

Since the unbiased estimate is satisfied in Kriging theory, *E*[*Z*^*∗*^ − *Z*] = 0, it can be obtained from equation (7) that

(8) $$\begin{array}{c} \overset{n}{\underset{i=1}{\sum }}\lambda_{i}=1\text{.} \end{array}$$

So, the variance of the estimation error can be expressed as

(9) $$\begin{array}{c} S=Var\left[Z^{*}\left(x_{0}\right)-Z\left(x_{0}\right)\right]=2\overset{n}{\underset{i=1}{\sum }}\lambda_{i}\gamma \left(x_{i}-x_{0}\right)-\overset{n}{\underset{i=1}{\sum }}\overset{n}{\underset{j=1}{\sum }}\lambda_{i}\lambda_{j}\gamma \left(x_{i}-x_{j}\right)\text{.} \end{array}$$

Since equation (9) needs to satisfy the unbiased estimation, a Lagrange multiplier needs to be introduced to solve the minimum variance of the estimation error, so equation (15) can be expressed as

(10) $$\begin{array}{c} S=2\overset{n}{\underset{i=1}{\sum }}\lambda_{i}\gamma \left(x_{i}-x_{0}\right)-\overset{n}{\underset{i=1}{\sum }}\overset{n}{\underset{j=1}{\sum }}\lambda_{i}\lambda_{j}\gamma \left(x_{i}-x_{j}\right)-2\mu \left(\overset{n}{\underset{i=1}{\sum }}\lambda_{i}-1\right)\text{.} \end{array}$$

Since equation (10) satisfies

(11) $$\begin{array}{c} \frac{\partial S}{\partial \lambda_{1}}=0,\ldots \ldots ,\frac{\partial S}{\partial \lambda_{n}}=0 \end{array}$$

therefore, we can get the following formula:

(12) $$\begin{array}{c} \overset{n}{\underset{i=1}{\sum }}\lambda_{i}\gamma \left(x_{i}-x_{0}\right)+\mu =\gamma \left(x_{j}-x_{0}\right)j=1,\ldots \ldots ,n\text{.} \end{array}$$

The estimated value *Z*^*∗*^(*x*_0_) can be calculated by the formula, and the variance *S* of the estimated error can be expressed as

(13) $$\begin{array}{c} S=\overset{n}{\underset{i=1}{\sum }}\lambda_{i}\gamma \left(x_{i}-x_{0}\right)+\mu \text{.} \end{array}$$

### 3.3. Random Field Modeling Method for Rotating Machinery Conditions

If it is assumed that there are *n* measurement points on the rotating machine and the spatial positions of the measurement points are *x*_*a*_(*a*=1,2,…, *n*), then the measured rotation parameters are *z*(*x*_*a*_), and the corresponding conditional random field *Z*_*c*_(*x*) is [19]

(14) $$\begin{array}{c} Z_{c}\left(x\right)=\left\{Z\left(x\right)|z\left(x_{a}\right),a=1,2,\ldots ,n\right\}\text{.} \end{array}$$

In order to obtain the final conditional random field *Z*_*c*_(*x*), it will be divided *Z*_*c*_(*x*) into two parts in space:

1. The position of *x*_*a*_(*a*=1,2,…, *n*), the known measurement point, and the measured parameter value *z*(*x*_*a*_).
2. Other unknown points on the rotating machine, which represent the known points: the number of information points represents the number of unknown information points, and *N* is the number of points in the conditional random field.

The conditional random field *Z*_*c*_(*x*) can then be expressed as

(15) $$\begin{array}{c} Z_{c}\left(x\right)=Z_{u}\left(x\right)+\left[Z_{k}\left(x\right)-Z_{s}\left(x\right)\right] \end{array}$$

where *Z*_*u*_(*x*) is the unconditional random field, *Z*_*k*_(*x*) is the field variable calculated by the optimal linear unbiased estimation based on the known spatial position*x*_*a*_(*a* = 1,2,…, *n*) of the measurement point and the measured parameter value*z*(*x*_*a*_), and *Z*_*s*_(*x*) is based on the spatial position *Z*_*u*_(*x*) of the unconditional random field and the corresponding parameter value. Field *x*_*a*_(*a* = 1,2,…, *n*) variables are calculated by optimal linear unbiased estimation.

Since *Z*_*k*_(*x*_*a*_)=*z*_*k*_(*x*_*a*_) and *Z*_*s*_(*x*_*a*_)=*Z*_*u*_(*x*_*a*_) are satisfied at each known measurement point position *x*_*a*_, the formula can be deduced to obtain

(16) $$\begin{array}{c} Z_{c}\left(x_{a}\right)=Z_{u}\left(x_{a}\right)+\left[z\left(x_{a}\right)-Z_{s}\left(x_{a}\right)\right]=z\left(x_{a}\right)\text{.} \end{array}$$

It can be seen from formula (14) that the conditional random field *Z*_*c*_(*x*) calculated according to formula (16) satisfies the measured parameter value *x*_*a*_ at each known measurement point position *z*(*x*_*a*_).

### 3.4. Rotating Machinery Condition Random Field Modeling Process and Steps

**Figure 2:**
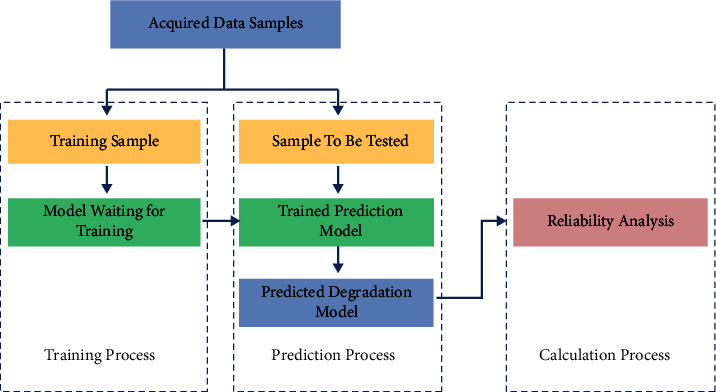
Flowchart of reliability analysis.

Generally speaking, in the data-based prediction model, the reliability of the rotating machinery after modeling according to the conditional random field can directly, accurately, and quickly reflect the working state of the rotating machinery [20], as shown in Figure 2. It can be divided into three stages. First, divide the acquired data into training samples and samples to be tested and use the training samples to mine data patterns to obtain a well-trained prediction model; then, by using the trained prediction model and the data to be tested, predict the expected degradation index of a certain rotating machinery; finally, the failure probability analysis or reliability analysis is carried out according to the degradation index. The relevant process is shown in Figure 2.

Correspondingly, the rotating mechanical condition random field is divided into five steps:

1. Firstly, random field theory is used to analyze the measured data of rotating machinery, and the uncertainty of rotating machinery is characterized, and the average value, standard deviation, and fluctuation range of rotating machinery parameters are obtained
2. The Kriging random field (known point) is obtained by using Kriging theory based on the known point survey data collected in practice *Z*_*k*_(*x*)
3. The unconditional random field *Z*_*u*_(*x*) is generated by the covariance matrix method through the parameters such as the mean value, standard deviation, fluctuation range, and autocorrelation function of the rotating machinery parameters obtained in step (1)
4. Based on the unconditional random field *Z*_*u*_(*x*) of the rotating machinery parameters obtained in step (3), the simulated values *Z*_*s*_(*x*) at the known points are obtained by Kriging theory
5. On the basis of the random field calculated in steps (2) to (4), the conditional random field *Z*_*c*_(*x*) of the rotating machinery parameters that conform to the actual engineering survey information can be obtained by the formula

## 4. Case Analysis

### 4.1. Project Background

**Figure 3:**
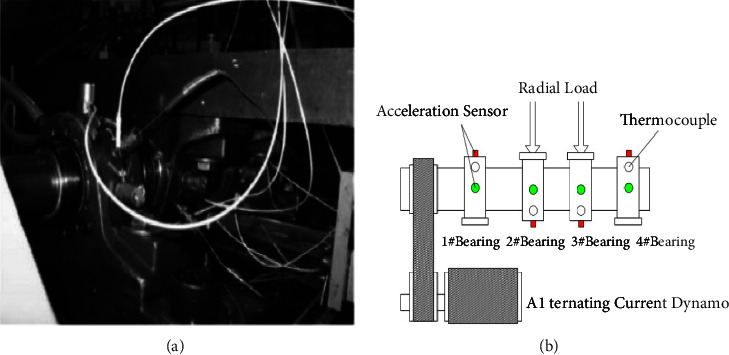
Schematic diagram of test machinery. (a) Test bench physical map. (b) Schematic diagram of test bench layout.

Cincinnati University Center for Intelligent Maintenance Systems (UC-IMS) published the vibration acceleration data of the rolling bearing life cycle [21] to establish a rotating machinery model based on conditional random fields. The layout of the rolling bearing test bench and sensor is shown in Figure 3. The four ZA-2115 double row roller bearings produced by Rexnord Company (bearing parameters are as follows: the number of rotors in each row is 16, and the roller diameter = 0.331 inch (i.e., 0.841 cm) and pitch diameter = 2.815 inch (i.e., 7.150 cm) are installed on the rotating shaft with a speed of 2000r/min. A radial load of 6000lbs (26.689 kN) is applied to the bearing and shaft through a spring mechanism. At the same time, a high-sensitivity ICP accelerometer (model: PCB353B33) was installed on the four bearing housings, and 20,480 data points were collected every 10 minutes at a sampling frequency of 20 kHz to realize the acquisition of vibration acceleration signals.

### 4.2. Establishment of Random Field of Rotating Machinery Condition Based on Measured Data

In this study, the whole life vibration acceleration data of 1#–4# bearings are used to establish a random field based on rotating machinery conditions. This group of experiments started and ended from 2004/02/12/10:32 to 2004/02/19/6:22, a total of 9840 min. During the experiment, the DAQCardTM-6062E data acquisition card of National Instruments was used to collect the vibration acceleration signal every 10 minutes. The length of each collected data point was 20480, and the total collected was 984 times. In the final stage of bearing operation, the metal structure of the outer ring falls off, which eventually leads to the failure of the bearing due to serious outer ring failure.

**Figure 4:**
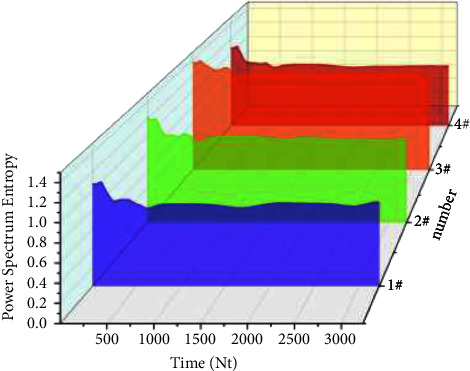
Power spectrum entropy of bearings.

It can be seen from Figure 4 that the power spectra of different bearings show the same trend with the increase of time, showing a trend of increasing first, then decreasing, and finally stabilizing. The decreasing fluctuation of the trend indicates that there are material differences between the bearings. The failure time points of 1–4 # bearings are located at the lowest point of their respective data change curves and are mostly located at the place with a time series of about 530. Because of the different properties of bearings, the dissipation energy of bearings 1 # and 4 # is relatively low because there is no radial shaft load, and the dissipation energy of bearings 2 # and 3 # is relatively high because there is radial shaft load. However, because there are certain differences among the bearings, the curves cannot completely coincide, Now, list the different parameters in Table 1.

Table 1 shows that the average crack depth of 1 #–4 # bearing is 5.82 mm, the average bearing speed is 895 rpm, the average input load is 5.5 nm, and the bearing failure time is 27.8. The coefficient of variation of these four parameters is less than 0.1, and the standard deviation is less than 0.5. Therefore, it is considered that these four parameters can be used as the characterization parameters of rotating machinery failure during the test. However, the calculation amount of establishing the conditional random field of rotating machinery with four parameters is complex. Therefore, after the parameters of 1 #–4 # bearing are statistically obtained, the Pearson correlation coefficient analysis can be used to obtain the correlation coefficient between the parameters, so as to obtain the most closely related parameters. The conditional random field can be established based on this parameter, which can simplify the calculation amount of establishing the conditional random field with little impact on the results.

**Figure 5:**
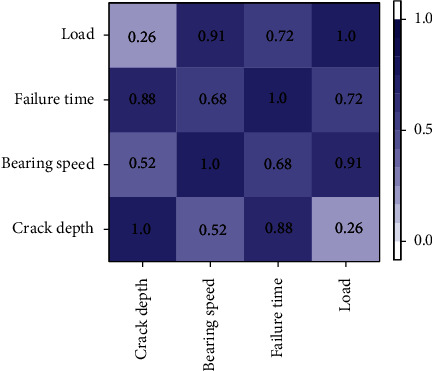
Pearson's correlation coefficient for each statistical parameter.

From Figure 5, it can be seen that the Pearson correlation coefficient between each parameter is greater than zero, so the all four parameters show a positive correlation trend. However, compared with the correlation between other parameters, the failure time is highly correlated with other parameters, so the failure time can be used to characterize other parameters. Therefore, the conditional random field of rotating machinery can be established according to the parameter of failure time. the failure time is positively correlated with other parameters, so the failure time is used to establish the conditional random field, refer to Section 3.4.

**Figure 6:**
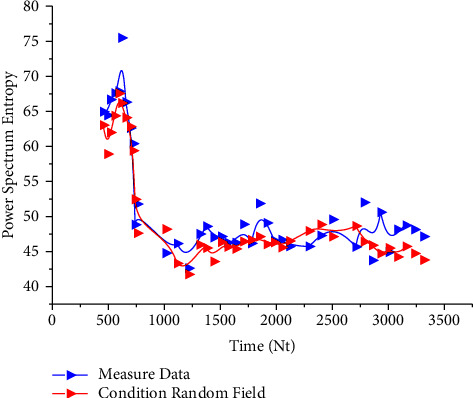
Failure time conditional random field of no. 1 bearing.

Figure 6 shows the conditional random field of the failure time of no. 1 bearing. It can be seen from Figure 6 that the data of the conditional random field of the failure time generated in this study and the failure time data measured by the bearing sampling have the same trend after removing some data outliers, and the values of the data points are relatively consistent.

**Figure 7:**
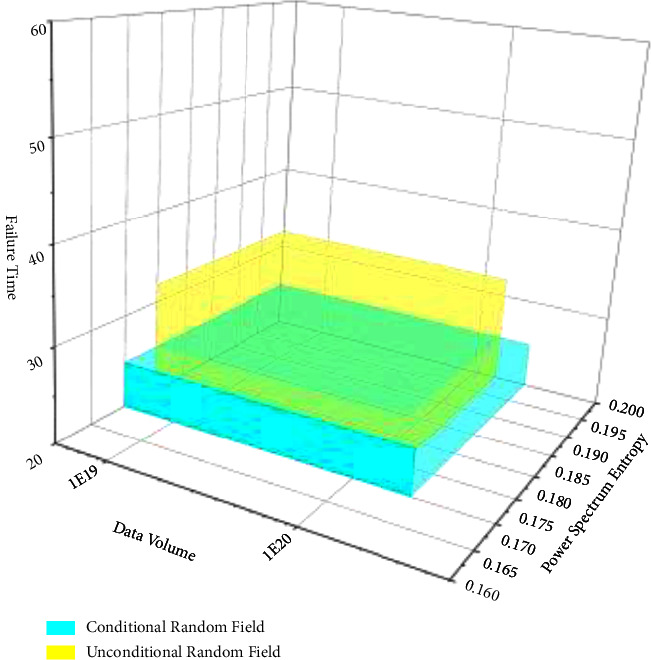
Failure time conditional random field.

After the establishment of the conditional random field based on the failure time of 1 # bearing, in order to verify the difference between the conditional random field and the nonconditional random field and the accuracy of data prediction by different random fields, the Markov model in Section 3.1 is now used as the model resume basis of the nonconditional random field, and the failure time of 1 #–4 # bearing is used to establish the conditional random field and the nonconditional random field, The comparison diagram is shown in Figure 7.

It can be shown from Figure 7 that the range of the data prediction model established by the conditional random field is larger than the model established by the nonconditional random field in the two dimensions of data sequence and power spectrum, and the range of the nonconditional random field is smaller than the model established by the nonconditional random field in the dimension of failure time. However, the failure time of the conditional random prediction is 25.6 h, which is closer to the measured data value than the 32.2 h predicted by the nonconditional random field. Therefore, the conditional random field established according to the failure time is closer to the real value than the predicted value of the nonconditional random section.

### 4.3. Reliability Analysis of Rotating Machinery

**Figure 8:**
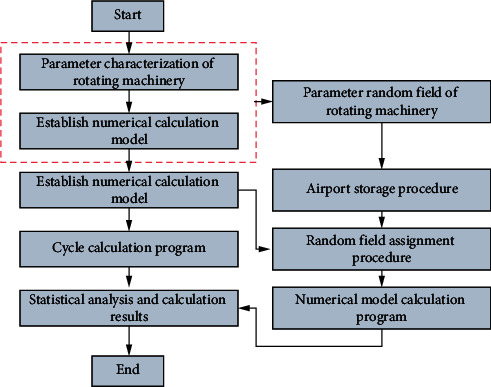
Reliability calculation process.

The vibration acceleration data of the rolling bearing in the whole life cycle disclosed by uc-ims is relatively complex; because in the process simulation of rotating machinery operation, considering the reliability analysis of rotating machinery, in order to simplify the simulation process and reduce the calculation workload as much as possible, only the failure time is used for analysis Matlab is used to analyze the reliability of rotating machinery. There are six main processes as shown in Figure 8:

1. Random field generation: based on the statistical characteristics of rotating machinery parameters, the random field files of relevant rock mass parameters are generated by MATLAB software.
2. The main program of calculation: this program mainly realizes the mutual call and automatic generation of subprograms. It is the core code of the random finite difference method calculation program.
3. Random field storage program: the main program calls the random field storage program, reads the random field file obtained by the random field generation program, and stores the random field of the rotating machine parameters in the finite difference software in the form of an array.
4. Random field assignment procedure: the random field stored in the random field storage program is assigned to the model unit through cell traversal by calling the random field assignment program by the main calculation program.
5. Numerical model calculation program: this part is a model calculation command flow written for rotating machinery objects. Users can modify this subroutine as required.
6. Circular calculation program: through the main calculation program, the circular calculation program of the model is automatically generated by the circular command, and the Monte Carlo simulation of the model is completed for hundreds of times. Based on the step-by-step decomposition method and the finite difference method, the generation of huge calculation code is avoided, and the storage space of the random finite difference method calculation program is saved.

In this studu, the reliability analysis of rotating machinery is carried out by using the failure probability calculation method in the literature. Based on the failure time samples of rotating machinery calculated 500 times, the probability distribution function of the failure time samples of rotating machinery is obtained by MATLAB software fitting.

**Figure 9:**
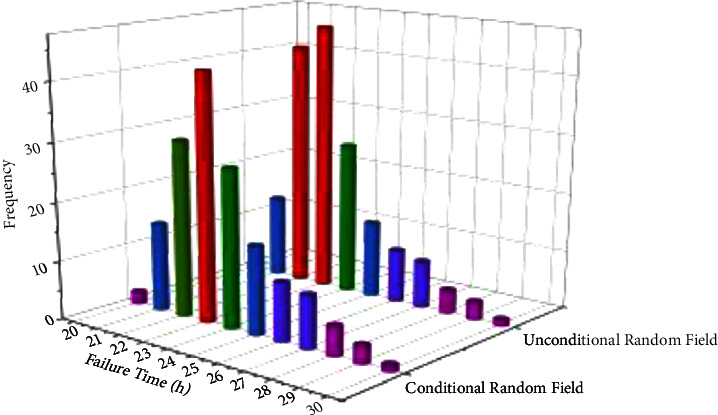
Histogram of failure time distribution of rotating machinery.

As shown in Figure 9, it can be seen that the maximum settlement value using the unconditional random field and the conditional random field follows the normal distribution. It can be seen that, after 500 Monte Carlo simulations, the average value and standard deviation of the maximum failure time sample value have converged. With the increase of Monte Carlo simulation, the probability distribution of the maximum settlement value of the tunnel vault will not change basically. The fitting function of the probability distribution of maximum failure time sample value is

(17) $$\begin{array}{c} f\left(S;\mu ,\sigma \right)=\frac{1}{s\sigma \sqrt{2\pi }}e^{{-\left(lns-\mu \right)}^{2}/{2\sigma }^{2}} \end{array}$$

where *S* is the maximum failure time of rotating machinery, *μ* is the sample mean value of the maximum failure time of rotating machinery obtained by fitting, and *σ* is the standard deviation of the maximum failure time sample of the rotating machinery obtained by fitting. The sample mean and standard deviation of the maximum failure time of the tunnel based on the unconditional random field and the conditional random field are shown in Table 2.

In order to analyze the failure probability of rotating machinery based on conditional random field, the calculation of tunnel failure probability of unconditional random field of rotating machinery under ordinary conditions is increased and compared with it. Monte Carlo simulation method based on hypercube Latin sampling method is adopted to establish the samples of conditional random field and unconditional random field of rotating machinery.

In Table 2, the failure probability of rotating machinery based on the unconditional random field *P*_*f*_ = 0.113%, and the reliability index *β* = 2.4527. The failure probability rotating machinery calculated based on the conditional random field generated from the borehole survey data is 0.019%, and the reliability index is *β* = 3.5678. When the conditional random field is used to describe the spatial variability of rock mass parameters of rotating machinery, the reliability index is increased by 0.8832.

**Figure 10:**
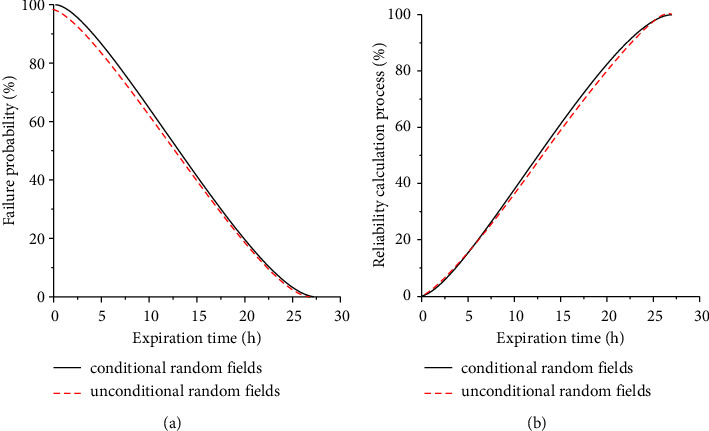
Failure probability distribution diagram. (a) Failure probability distribution. (b) Cumulative probability distribution.

Figure 10 shows the failure probability distribution and cumulative probability distribution of rotating machinery. For the failure time threshold of rotating machinery, the tunnel failure probability calculated based on the conditional random field is smaller than the failure probability of rotating machinery calculated based on the unconditional random field. Therefore, in the reliability analysis of actual rotating machinery, the random field theory cannot be simply used to describe the spatial variability of rotating machinery. The random field corresponding to the site should be generated based on the known information; otherwise, the calculation result will be too large.

## 5. Conclusion

Taking the database of Cincinnati University as the source of data as the engineering background, this study establishes the method of generating conditional random fields describing rotating machinery and introduces the generated conditional random fields into the numerical calculation model of rotating machinery. Based on the Monte Carlo simulation results, the reliability index of rotating machinery is calculated according to the reliability theory. The following conclusions are obtained through the research:

1. The conditional random field generation method based on Kriging theory can describe the spatial variability of rotating machinery parameters efficiently. The stochastic finite difference calculation program can effectively avoid the huge calculation code in the traditional stochastic finite difference calculation program and greatly save the storage space;
2. In the analysis of rotating machinery in the database of Cincinnati University, the failure probability during rotation is only 0.019%, and the reliability index *β* = 3.3359, meeting the construction requirements of the project;
3. The failure probability of rotating machinery calculated by the conditional random field generated based on the measured information is smaller than the tunnel failure probability calculated by the conventional nonconditional random field generated without considering the survey information. When the data in the site are considered, the failure probability of rotating machinery based on the unconditional random field *P*_*f*_ = 0.113%, and the reliability index *β* = 2.4527. The failure probability rotating machinery calculated based on the conditional random field generated from the borehole survey data is 0.019%, and the reliability index is *β* = 3.5678. The reliability index of the failure time of rotating machinery is increased by 0.8823.

## Figures and Tables

**Table 1:** Rotating machinery statistical parameters.

Number	Parameter	Crack depth (mm)	Bearing speed (rpm)	Load (Nm)	Failure time (h)
1–4#	Average	5.82	895	5.5	27.8
	Standard deviation	0.33	0.16	0.05	0.28
	Coefficient of variation	0.25	0.48	0.11	0.34

**Table 2:** Reliability index of different random fields.

Field type	Failure time of rotating machinery (h)		Failure probability	Reliability index (*β*)
	Mean (*μ*)	Standard deviation (*σ*)		
Unconditional random field	27.8944	2.5984	0.108	2.4527
Conditional random field	26.8753	2.2558	0.019	3.3359

## Data Availability

The experimental data used to support the findings of this study an be obained from the corresponding author upon request.
